# Prosthetic Correction of Proclined Maxillary Incisors: A Biomechanical Analysis

**DOI:** 10.1155/2019/7416076

**Published:** 2019-07-09

**Authors:** Yiting He, Yung-Chung Chen, Wei Teng, Alex S. L. Fok, Hooi Pin Chew

**Affiliations:** ^1^Department of Prosthodontics, Hospital of Stomatology, Sun Yat-sen University, Guangdong Provincial Key Laboratory of Stomatology, Guangzhou 510016, China; ^2^Institute of Oral Medicine, College of Medicine, National Cheng Kung University, Tainan, Taiwan; ^3^Minnesota Dental Research Center for Biomaterials and Biomechanics, School of Dentistry, University of Minnesota, Minneapolis, MN, USA

## Abstract

In some cases of proclined maxillary incisors, the proclination can be corrected by a fixed prosthesis. The aim of this study was to investigate the magnitude and distribution of (i) principal stresses in the adjacent alveolar bone and (ii) direct and shear stresses that are normal and parallel, respectively, to the bone-tooth interface of a normal angulated maxillary incisor, a proclined one, and a proclined one corrected with an angled prosthetic crown. 2D finite-element models were constructed, and a static load of 200 N on the palatal surface of the maxillary incisor at different load angles was applied. Load angles (complementary angle to interincisal angle) ranging from 20° to 90° were applied. The results indicate that the load angle could have a more significant impact on the overall stress distributions in the surrounding alveolar bone and along the bone-tooth interface than the proclination of the maxillary incisor. Provided that the resulting interincisal angle is 150° or smaller, the stresses in the surrounding bone and at the bone-tooth interface are similar between a proclined maxillary incisor and the one with prosthodontic correction. Hence, such a correction, when deemed appropriate clinically, can be undertaken with confidence that there is little risk of incurring additional stresses over that already in existence, in the supporting bone and at the tooth-bone interface.

## 1. Introduction

Facial appearance, function, and competence of the lips are greatly influenced by the alignment (or malalignment) of the maxillary incisor, and its protrusion, especially, has a negative impact on appearance and psychosocial wellbeing. In addition, excess overjet and overbite due to protrusion may also increase the vulnerability of the front teeth to injury and periodontal damage. Thus, the index of orthodontic treatment need (IOTN), which defines the severity of malocclusion and priority for treatment, considers excess overjet the second worst type of malocclusion [1]. Often, no other malocclusion occurs except for a lingually or labially displaced incisor, and if a simple labiolingual displacement, i.e., protrusion, is involved, a removable appliance is used to correct the protrusion. If the incisors are rotated or tipped, fixed orthodontic appliance therapy is indicated [2]. Orthodontic intervention for protruded maxillary incisors is always best performed during the period of a child's or adolescence's growth spurt for optimum results. However, the assessment of the need for treatment differs greatly among practitioners [3], and not all patients are able to receive timely and appropriate orthodontic intervention. As the demand for esthetics and function of speech and social interaction increases with age, these patients may seek treatment to correct the malalignment of their front teeth when they become adults [4]. In less severe malocclusion as mentioned above, to minimize time and cost, a prosthodontic-centered approach may be proposed [5, 6], and the correction of overjet or overbite can be accomplished by replacing the natural crown with an angled prosthesis [7]. However, the effect of this procedure on the mechanical stresses in the tissues surrounding the corrected tooth has not been studied in detail.

The aim of this study is therefore to perform finite-element (FE) analysis to compare the stress distributions in the supporting tissues, especially those at the bone-tooth interface, between a normal angulated maxillary incisor, a proclined one, and a proclined one with an angle-corrected prosthetic crown. The results will help determine whether such prosthodontic correction is a viable treatment option for proclined maxillary incisors.

## 2. Materials and Methods

The finite-element method is a numerical tool ideal for analyzing the mechanical behavior of manmade and biological structures, including their combinations. It can help us better understand the potential risks or benefits of a dental treatment such as the aforementioned angle correction of a protruded maxillary incisor using a prosthetic crown.

In this study, linear-elastic static analyses were performed to calculate the stress distributions in the surrounding bone and at the bone-tooth interface for a maxillary incisor during occlusion, with or without malalignment, and in the former, with or without angle correction.

2D rather than 3D finite-element models will be used in this study as it has been found previously that the numerical differences observed between 2D and 3D analyses of dental restorations in a single tooth unit are small [8]. Moreover, the complexity of 3D FE models often makes it impossible to achieve the same mesh refinement and hence numerical accuracy as in 2D models. 3D models that involve a periodontal ligament (PDL) that is much thinner than the surrounding structures may produce highly distorted elements and hence unreliable numerical results. For simplicity, the endodontic treatment that may be required for the correction was not considered.

### 2.1. FE Models

**Figure 1:**
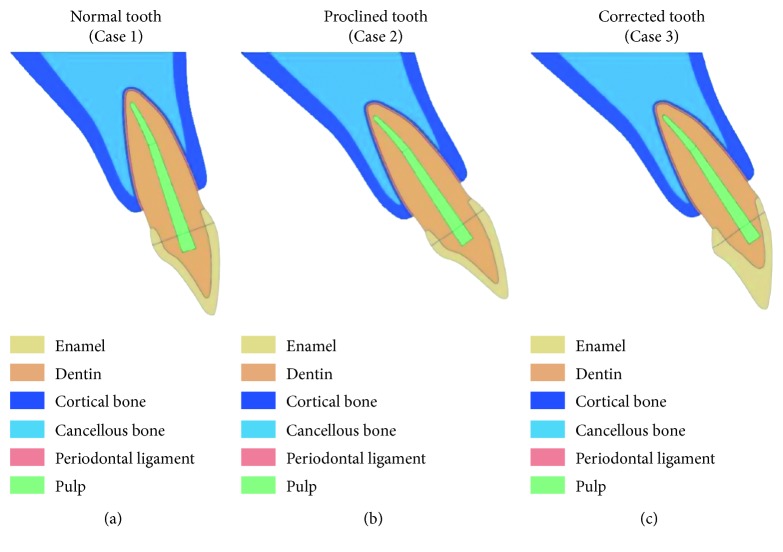
Models for (a) normal tooth (Case 1), (b) proclined tooth (Case 2), and (c) corrected tooth (Case 3).

Two-dimensional finite-element models of a human maxillary central incisor and the surrounding PDL and bone were constructed by using the software SolidWorks (version 2016; Dassault Systèmes SOLIDWORKS Corp, Waltham, MA, USA) based on data in the literature [7]. The inclination of the maxillary incisor is conventionally determined by the angle of the long axis of the tooth relative to the maxillary plane [9]. The average angulation of a maxillary incisor to the maxillary plane for Caucasians is reported to be 109° ± 6° (mean ± SD) [10]. We therefore used 110° to represent the norm (Case 1). To simulate a proclined maxillary incisor, we adopted an angle of 125° (Case 2). The shape of the bone for Case 2 was modified in order to adapt to the increased inclination of the maxillary incisor while keeping the distance between the crest of the alveolar bone and the cement-enamel junction to 2 mm. The oral mucosa surrounding the bone was not included as it does not offer much resistance to the occlusal load. The model with prosthodontic treatment (Case 3) was generated by combining the inclination of the crown in Case 1 and that of the root in Case 2 to simulate a proclined maxillary incisor corrected with a fixed prosthesis at a normal inclination (Figure 1). The models were exported to ABAQUS (version 6.11; Dassault Systèmes Simulia, Waltham, MA, USA) for stress analysis.

### 2.2. Material Properties and Loading Conditions

The mechanical properties of the materials were considered to be linearly elastic, isotropic, and homogeneous. The material properties of each part are shown in Table 1. The models were meshed with quadrilateral and triangular plane-strain elements (CPE4R and CPE3) [16]. A 10 mm out-of-plane thickness was applied to the models. The connection between the alveolar bone and the tooth was set as the surface-to-surface tied contact [17]. This allowed the direct and shear stresses that are normal and parallel, respectively, to the interface to be determined. The number of nodes at the bone-tooth interface was the same for all three models to ease comparison. The cranial base was fully fixed.

**Figure 2:**
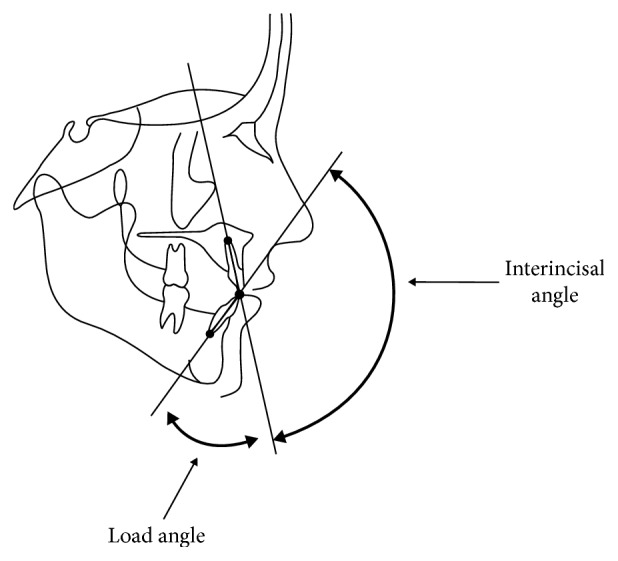
Cephalometric diagram illustrating the interincisal angle and load angle (modified from Figure 1 in [18]).

In the present study, the direction of the load was assumed to follow the long axis of the lower incisor with the load angle being the complementary angle to the interincisal angle (Figure 2). The interincisal angle measures the external angle subtended by the long axes of the maxillary and mandibular central incisors (Figure 2). It determines the degree of labial inclination or proclination of the incisors. The more proclined the incisors, the lower the interincisal angle. The average interincisal angle is 135° ± 10^9^. Class II division 1 cases of incisor relationships usually have a smaller than average interincisal angle, whereas Class II division 2 cases usually have a larger than average interincisal angle. In order to simulate a range of interincisal angles, load angles ranging from 20° to 90° were considered.

**Figure 3:**
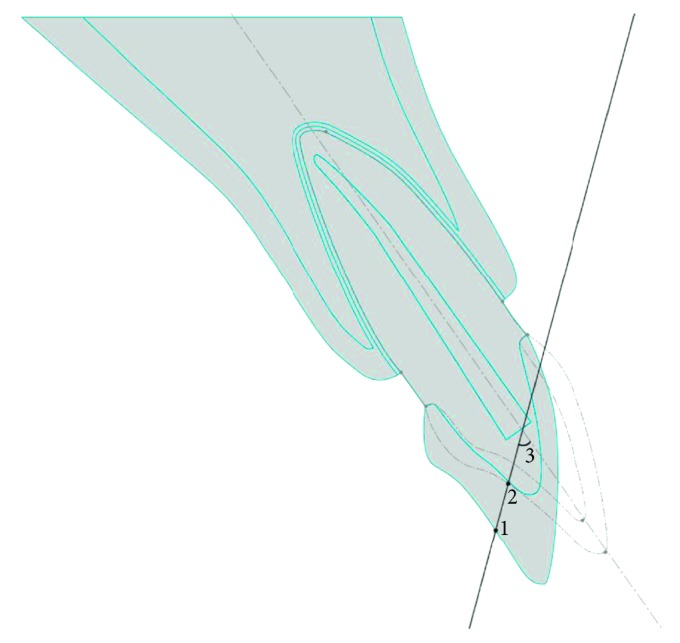
Positions of the load in Cases 2 and 3. Point 1: load position in corrected tooth. Point 2: load position in proclined tooth. Angle 3: angle subtended by the load vector with respect to the long axis of the tooth.

A concentrated load of 200 N was applied to simulate the occlusal force. The position of the load for Case 1 was 3 mm above the incisal edge, corresponding to an overjet of 2 mm. An overjet of 4.75 mm was assumed for Case 2, which corresponded to a moderate need for orthodontic treatment (3.5–6.0 mm) [19]. For Case 3, the position of the load was determined from the intersection of the load vector in Case 2 and the labial contour of the crown in Case 3 (Figure 3).

### 2.3. Evaluation of Stresses in Supporting Tissues and at Their Interfaces

Principal stresses within the cortical and the cancellous bone and the normal and shear stresses at the bone-tooth interface were assessed.

For ease of comparison between the three cases, the interfacial stresses were averaged over all the nodes along the interface. For the interfacial normal stresses, the tensile and compressive values were considered separately and in combination (by averaging the absolute values). For the interfacial shear stresses, only the absolute values were considered as the sign or direction of a shear stress was not important as far as the biomechanical response was concerned.

## 3. Results

### 3.1. Stress Distributions within the Surrounding Bone

**Figure 4:**
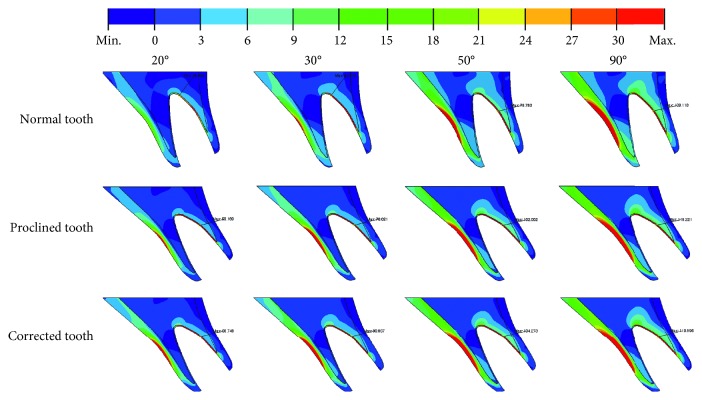
Maximum principal stress distributions in the alveolar bone under 20°, 30°, 50°, and 90° load angles for Cases 1, 2, and 3.

**Figure 5:**
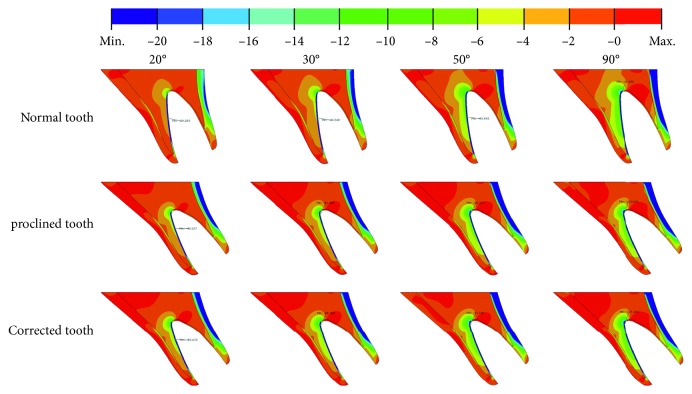
Minimum principal stress distributions in the alveolar bone under 20°, 30°, 50°, and 90° load angles for Cases 1, 2, and 3.

The alveolar bone is subjected to both axial compression and a counterclockwise bending moment. The larger the load angle, the larger the bending moment and the smaller the axial compression (Figure 2). Figures 4 and 5 show, respectively, the maximum and minimum principal stress distributions within the bone. The labial side of the outer cortical bone is under compression, while the palatal side is under tension. The opposite is true for the cancellous bone. Figures 4 and 5 show that there are stress concentrations in the cortical bone, especially in the thin layer surrounding the tooth socket (lamina dura), with a magnitude greater than 100 MPa. The magnitude of the stresses increases when the load angle changes from 20° to 90° for all three cases.

### 3.2. Stresses at the Bone-Tooth Interface

**Figure 6:**
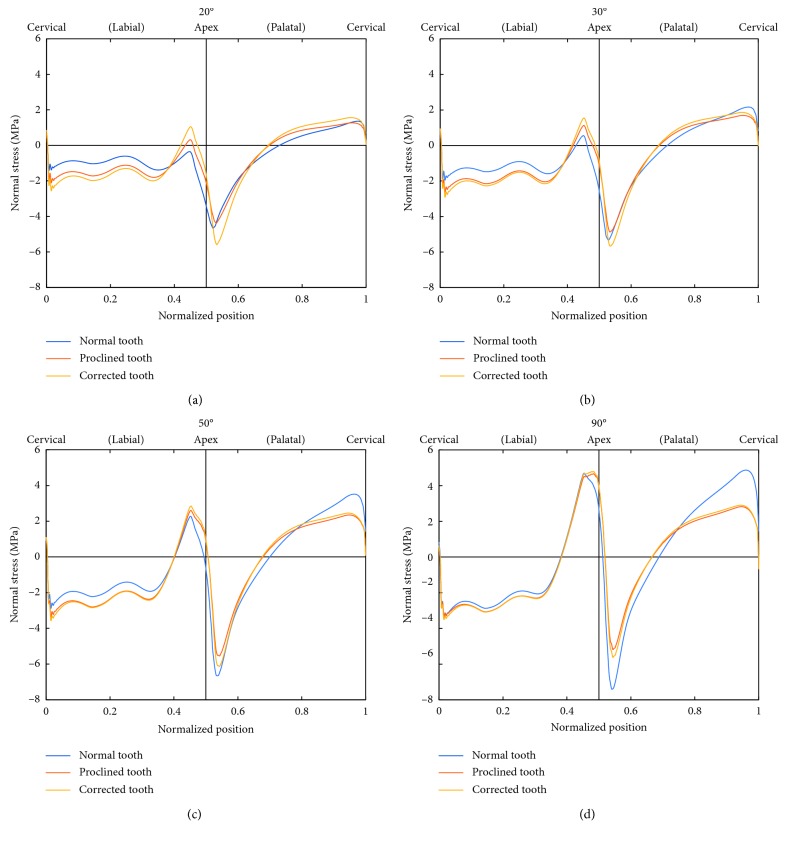
Distributions of stress normal to the bone-tooth interface for Cases 1, 2, and 3 at 20° (a), 30° (b), 50° (c), and 90° (d) load angles.

**Figure 7:**
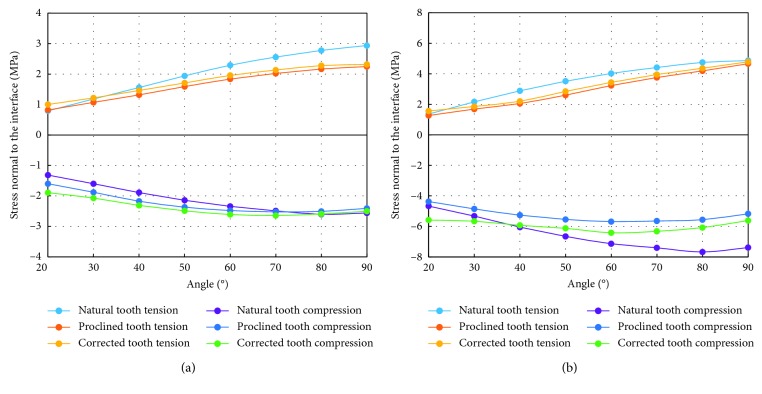
Average (a) and maximum (b) of the normal stresses (tensile and compressive) along the bone-tooth interface at different load angles.

**Figure 8:**
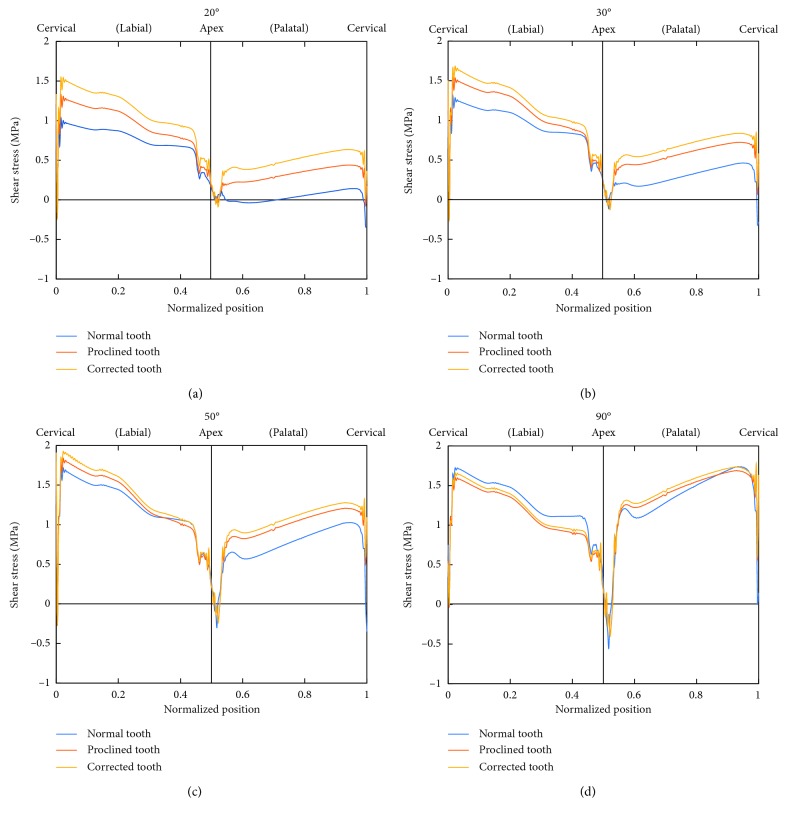
Distributions of shear stress at the bone-tooth interface for Cases 1, 2, and 3 at 20° (a), 30° (b), 50° (c), and 90° (d) load angles.

**Figure 9:**
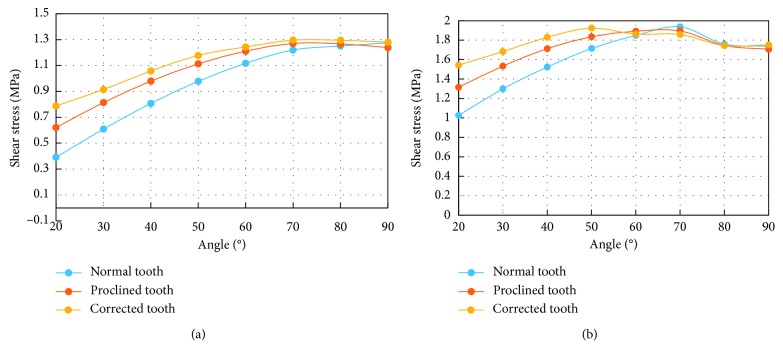
Average (a) and maximum (b) of the shear stresses along the bone-tooth interface at different load angles.

The magnitudes of the stresses along the tooth-bone interface are much lower than those in the bone (Figures 6–9).

Figure 6 compares the direct stress normal to the bone-tooth interface between Cases 1, 2, and 3 for load angles of 20°, 30°, 50°, and 90°. The stresses are plotted against the normalized distance along the interface, starting from the labial alveolar crest (cervical), moving towards the apical end of the tooth socket, and ending at the palatal alveolar crest.

The direct stress normal to the bone-tooth interface on the labial tooth socket is mostly compressive but becomes tensile towards the apex where there is stress concentration. It then changes sharply to being compressive on the palatal side of the apex before reversing back to being tensile towards the alveolar crest. The stresses on the palatal side are generally higher than those on the labial side in Case 1 (normal tooth), but the opposite is true for Cases 2 and 3 (proclined tooth and corrected tooth). The distribution and magnitude of the normal stress in Cases 2 and 3 are similar to increasing load angle, except at the apical area under the smaller load angles of 20° and 30° where the stresses in Case 3 are higher than those in Case 2. The magnitude of the interfacial normal stress at both the labial and palatal sides of the apex increases with increasing load angle for all three cases, with the increase on the palatal side of the apex for Case 3 being the least pronounced.

The average tensile and compressive interfacial stresses increase with the load angle, with the average tensile stress increasing faster than the average compressive stress (Figure 7(a)). Also, the stresses in the normal tooth increase faster with the load angle than those of the other two cases. Consequently, although it has lower stresses at the smaller load angles, its stresses exceed those of the other two cases at larger load angles. The maximum tensile and compressive interfacial stresses show similar trends as the average stresses (Figure 7(b)), except that the values in the normal tooth exceed those in the other two cases at small load angles.

Table 2 shows the average of the absolute values of the normal stresses over all the nodes along the bone-tooth interface for all load angles. Similar trends as those found for the individual average of the tensile and compressive stresses can be seen. When averaged over all the load angles, it can be seen that all three cases have similar stress values (∼2.0 MPa), with the difference being less than 7%.

The shear stress at the bone-tooth interface is minimal at the apical area and is mainly present on the labial and palatal sides of the tooth socket (Figure 8). Its magnitude also increases with increasing load angle. However, its peak value (∼2 MPa) is much lower than that of the normal stress (∼8 MPa). At small load angles, the labial side has higher interfacial shear stresses than the palatal side. As the load angle increases, the shear stress on the palatal side increases faster than that on the labial side, resulting in similar magnitudes on both sides of the root at larger load angles. Figure 8 compares the shear stress along the bone-tooth interface between Cases 1, 2, and 3 for load angles of 20°, 30°, 50°, and 90°. The distribution and magnitude of the shear stress in Cases 2 and 3 are again similar at large load angles. At the smaller load angles of 20° and 30°, the stresses in Case 3 are larger than those in Case 2.

The average of the absolute values of the shear stress over all the nodes along the bone-tooth interface (Figure 9(a)) exhibits a trend, against the load angle, similar to the average normal compressive stress (Figure 7(a)). The three cases show more differences at small load angles but converge to similar values as the load angle increases. The maximum interfacial shear stress shows a similar trend, except that the convergence occurs earlier (Figure 9(b)). The average value over all the load angles, however, shows larger differences between the cases, with that of Case 1 (0.97 MPa) being 10% and 17% lower than those of Case 2 (1.07 MPa) and Case 3 (1.14 MPa), respectively (Table 3).

## 4. Discussion

This study evaluated the effect of prosthodontic correction on the stresses of the bone surrounding a proclined maxillary incisor using the FE method. Some previous FE analysis investigating the effect of maxillary incisor proclination suggested that the bigger the proclination of the incisor, the higher the stress at the root apex. External root resorption has been shown to occur when stresses induced by intrusion at the apex exceed the resistance and reparative ability of periapical tissues [20]. However, these studies focused on labial forces applied to cause orthodontic movement instead of chewing forces on the lingual surface considered in the present study [21].

The results of this study indicate that, during chewing, the load angle, and hence the interincisal angle, could have a more significant impact on the overall stress distributions in the surrounding bone and along the bone-tooth interface than the proclination angle. Considering the Class I interincisal edge relationship and the position of loading according to the average overjet distance [17], the load angle used ranged from that which is perpendicular to the maxillary plane to that perpendicular to the long axis of the maxillary incisor. The lowest stresses were found when load was applied almost parallel to the long axis of the maxillary incisor. Surprisingly, perhaps, the normal maxillary incisor was found to have higher stresses at the larger load angles than the proclined tooth, with or without correction. This is because for the proclined maxillary incisor, the point of contact with the lower incisor is further away from the incisal edge, thus reducing the bending moment about the apex and hence the resulting stresses.

The present study shows that the stresses in the surrounding tissues of the proclined incisors, with and without prosthodontic correction, are similar except at the apical area for the smaller load angles of 20° and 30° (Figures 6 and 8), where the magnitude of stresses in the corrected incisor is higher than that without correction. Hence, in order to maintain physiologically tolerable stresses in the surrounding tissues, prosthetic corrections of proclined upper incisors where the interincisal angle is larger than 150° should be done with caution. Otherwise, such a treatment option for proclined maxillary incisors appears to be viable.

The normal and shear stresses along the tooth-bone interface were analyzed separately as they may be responsible for different modes of biomechanical responses. Furthermore, the tensile and compressive normal stresses were considered separately. This is because the strength of the bone depends on the mode of loading [22]. The range of loading (stress × time) for the maintenance of bone density has been reported [23]. However, the magnitude and duration of stresses responsible for tooth movement, reduced periodontal health, and tooth and bone resorption are highly case dependent. The stresses calculated in this study were therefore used for comparison between different cases only, with the sole aim of assessing the viability of prosthodontic correction for proclined maxillary incisors. Nevertheless, it should be pointed out that some of the stresses, especially those in the thin cortical layer surrounding the tooth socket, i.e., the lamina dura, were probably overestimated (they are close to the measured cortical bone strength) [22]. This is attributed to the geometrical simplifications of the models used in this study, which did not account for the full resistance to bending of the cylindrical lamina dura. The graded distribution of bone density was also not considered—an abrupt change in assumed mechanical properties will lead to stress concentrations in the model [24].

During biting or chewing, the lower incisor moves along the occlusal surface of the upper one, changing both the direction and position of contact. If a bolus of food is considered, the tooth-loading process would be more complicated. These are things that have not been considered in this study. In addition, the current model did not take into consideration endodontically treated dentine and the usage of a post, as the scope of this study was to study the distribution of stresses in the supporting tissues. Indeed, clinically, failure can occur in the restoration used to correct the proclination. Effects on the patient's mastication of the prosthodontic correction will also need to be assessed. Further studies will need to be conducted on the impact of the above scenario on the stress distributions of the supporting tissues, the endodontically treated tooth, and the prosthodontic crown used to correct the angle.

## 5. Conclusion

Provided that the resulting interincisal angle is 150° or smaller, the stresses in the surrounding bone and at the bone-tooth interface are similar between a proclined maxillary incisor and the one with prosthodontic correction. Hence, such a correction, when deemed appropriate clinically, can be undertaken with confidence that there is little risk of incurring additional stresses over that already in existence, in the supporting bone and at the tooth-bone interface.

## Figures and Tables

**Table 1:** Material properties used in the FE models.

	Young's modulus (MPa)	Poisson's ratio	Ref.
Enamel	84100	0.3	[11]
Dentin	18600	0.33	[12]
Cortical bone	13700	0.33	[13]
Cancellous bone	1370	0.33	[13]
Periodontal ligament	6.89	0.45	[14]
Pulp	2.07	0.45	[15]

**Table 2:** Average of the absolute values of stresses (MPa) normal to the bone-tooth interface at different load angles.

	20°	30°	40°	50°	60°	70°	80°	90°	Avg.
Normal tooth	1.1700	1.4616	1.7678	2.0634	2.3207	2.5200	2.6808	2.7289	2.0891
Proclined tooth	1.3394	1.5739	1.8151	2.0283	2.1913	2.2934	2.3497	2.3322	1.9904
Corrected tooth	1.5664	1.7347	1.9526	2.1453	2.3194	2.4115	2.4479	2.4163	2.1243

**Table 3:** Average of the absolute values of shear stresses (MPa) along the bone-tooth interface at different load angles.

	20°	30°	40°	50°	60°	70°	80°	90°	Avg.
Normal tooth	0.4052	0.6167	0.8152	0.9863	1.1268	1.2316	1.2598	1.2849	0.9658
Proclined tooth	0.6233	0.8168	0.9836	1.1187	1.2179	1.2790	1.2757	1.2479	1.0704
Corrected tooth	0.7904	0.9176	1.0627	1.1841	1.2492	1.3034	1.3043	1.2903	1.1378

## Data Availability

The finite-element model data used to support the findings of this study are available from the corresponding author upon request.

## References

[1] Avinash B., Shivalinga B. M., Shekar S. (2015). The index of orthodontic treatment need- a review of orthodontics JSS DC and Hospital Mysore. *International Journal of Recent Scientific Research*.

[2] Schupak G. E., Hung J., McNulty E. C. (2014). Esthetics and orthodontics. *Esthetic Dentistry*.

[3] Jawad Z., Bates C., Hodge T. (2015). Who needs orthodontic treatment? Who gets it? and who wants it?. *British Dental Journal*.

[4] Neely M. L., Miller R., Rich S. E., Will L. A., Wright W. G., Jones J. A. (2017). Effect of malocclusion on adults seeking orthodontic treatment. *American Journal of Orthodontics and Dentofacial Orthopedics*.

[5] Xie C., Meng Y. (2016). A case report of esthetic and functional correction of maxillary protrusion using a prosthodontics-centered multidisciplinary approach. *International Journal of Periodontics and Restorative Dentistry*.

[6] Singh K., Kumar N., Choudhary N., Gupta N. (2013). Unconventional prosthodontics for the aesthetic rehabilitation of discoloured rotated maxillary central incisor. *BMJ Case Reports*.

[7] Nelson S. J., Nelson S. J. (2014). The permanent maxillary incisors. *Wheeler’s Dental Anatomy, Physiology and Occlusion*.

[8] Romeed S. A., Fok S. L., Wilson N. H. F. (2006). A comparison of 2D and 3D finite element analysis of a restored tooth. *Journal of Oral Rehabilitation*.

[9] Gill D. S., Naini F. B., Newell D. B. (2011). Diagnosis and treatment planning. *Orthodontics: Principles and Practice*.

[10] Mitchell L., Nelson-Moon Z. L., Mitchell L. (2013). Cephalometrics. *An Introduction to Orthodontics*.

[11] Farah J. W., Craig R. G., Meroueh K. A. (1989). Finite element analysis of three- and four-unit bridges. *Journal of Oral Rehabilitation*.

[12] Kinney J. H., Marshall S. J., Marshall G. W. (2003). The mechanical properties of human dentin: a critical review and re-evaluation of the dental literature. *Critical Reviews in Oral Biology and Medicine*.

[13] Wall A., Board T. (2014). The compressive behavior of bone as a two-phase porous structure. *Classic Papers in Orthopaedics*.

[14] Yettram A. L., Wright K. W. J., Houston W. J. B. (1977). Centre of rotation of a maxillary central incisor under orthodontic loading. *British Journal of Orthodontics*.

[15] Couegnat G., Fok S. L., Cooper J. E., Qualtrough A. J. E. (2006). Structural optimization of dental restorations using the principle of adaptive growth. *Dental Materials*.

[16] Dassault Systèmes Simulia (2012). *Analysis User’s Manual Volume 4, Abaqus 6.12*.

[17] Dassault Systèmes Simulia (2012). *Analysis User’s Manual: Volume 5: Prescribed Conditions, Constraints and Interactions, Abaqus 6.12, Vol. IV*.

[18] Gijbels F., Serhal C. B., Willems G. (2001). Diagnostic yield of conventional and digital cephalometric images: a human cadaver study. *Dentomaxillofacial Radiology*.

[19] Brook P. H., Shaw W. C. (1989). The development of an index of orthodontic treatment priority. *European Journal of Orthodontics*.

[20] Dermaut L. R., De Munck A. (1986). Apical root resorption of upper incisors caused by intrusive tooth movement: a radiographic study. *American Journal of Orthodontics and Dentofacial Orthopedics*.

[21] Choi S.-H., Kim Y.-H., Lee K.-J., Hwang C.-J. (2016). Effect of labiolingual inclination of a maxillary central incisor and surrounding alveolar bone loss on periodontal stress: a finite element analysis. *The Korean Journal of Orthodontics*.

[22] Hansen U., Zioupos P., Simpson R., Currey J. D., Hynd D. (2008). The effect of strain rate on the mechanical properties of human cortical bone. *Journal of Biomechanical Engineering*.

[23] Crupi V., Guglielmino E., La Rosa G., Vander Sloten J., Van Oosterwyck H. (2004). Numerical analysis of bone adaptation around an oral implant due to overload stress. *Proceedings of the Institution of Mechanical Engineers, Part H: Journal of Engineering in Medicine*.

[24] Chen Y., Fok A. (2014). Stress distributions in human teeth modeled with a natural graded material distribution. *Dental Materials*.

